# Differential Mental Health Impact Six Months After Extensive River Flooding in Rural Australia: A Cross-Sectional Analysis Through an Equity Lens

**DOI:** 10.3389/fpubh.2019.00367

**Published:** 2019-12-06

**Authors:** Veronica Matthews, Jo Longman, Helen L. Berry, Megan Passey, James Bennett-Levy, Geoffrey G. Morgan, Sabrina Pit, Margaret Rolfe, Ross S. Bailie

**Affiliations:** ^1^The University of Sydney, University Centre for Rural Health, Lismore, NSW, Australia; ^2^Centre for Health Systems and Safety Research, Macquarie University, Sydney, NSW, Australia

**Keywords:** natural disasters, mental health, inequality, indigenous populations, low income populations

## Abstract

**Background:** Northern New South Wales in Australia is a “hotspot” for natural disaster declarations with recent extensive flooding in early 2017. With limited knowledge about how climate change affects mental health and resilience, robust local assessments are required to better understand long-term impact, particularly in communities prone to extreme weather events.

**Methods:** Six months post-flood, a cross-sectional survey of adults living in the region during the flood was conducted to quantify associations between flood impact and psychological morbidity (post-traumatic stress (PTSD), anxiety, depression, suicidal ideation) for different exposure scenarios, and respondent groups. We adopted a community-academic partnership approach and purposive recruitment to increase participation from marginalized groups.

**Results:** Of 2,180 respondents, almost all (91%) were affected by some degree of flood-related exposure at an individual and community level (ranging from suburb damage to home or business inundated). Socio-economically marginalized respondents were more likely to have their homes inundated and to be displaced. Mental health risk was significantly elevated for respondents: whose home/business/farm was inundated [e.g., home inundation: PTSD adjusted odds ratio (AOR) 13.72 (99% CI 4.53–41.56)]; who reported multiple exposures [e.g., three exposures: PTSD AOR 6.43 (99% CI 2.11–19.60)]; and who were still displaced after 6 months [e.g., PTSD AOR 24.43 (99% CI 7.05–84.69)].

**Conclusion:** The 2017 flood had profound impact, particularly for respondents still displaced and for socio-economically marginalized groups. Our community-academic partnership approach builds community cohesion, informs targeted mental health disaster preparedness and response policies for different sectors of the community and longer-term interventions aimed at improving community adaptability to climate change.

## Introduction

There is compelling evidence and wide consensus that anthropogenic activities are causing climate change, leading to more frequent extreme weather events with adverse consequences for public health, disproportionately so for the poorest populations (1, 2). In academic and public discourse on health impacts from climate change, connection to mental health has generally been neglected (3). Robust and context-specific case-studies assessing extreme weather and mental health are therefore required to strengthen the case for effective adaptation, particularly in community settings, and incorporating the experience of diverse socio-economic groups (4, 5).

Risk of climate change effects and adverse impacts are known to exacerbate existing inequalities in all countries regardless of their level of development (1, 6). Landmark international agreements, such as the United Nations' Agenda 2030 (Sustainable Development Goals) and the Sendai Framework for Disaster Risk Reduction (2015–2030), recognize the need for complementary action on climate change mitigation and adaptation, with inequality a key global challenge to creating sustainable and resilient communities (7, 8). The Sendai Framework advocates a community-centered preventive approach to disaster risk. It recommends that government agencies be multi-sectoral and inclusive in designing and implementing policies by engaging all relevant stakeholders, including women, children, seniors, people with pre-existing health conditions, people with low socio-economic status and Indigenous communities. In this way, understanding and managing disaster risk encompasses all dimensions of exposure, vulnerability, and capacity of individuals and communities in formulating regional and local risk reduction policies (8).

Floods are the most expensive weather-related event in Australia with an average annual damage bill of over $300 million (9). Such annual assessments do not regularly incorporate costs from less visible social impacts (e.g., mental health and well-being or employment), nor how impacts are differentially distributed amongst societal groups. While river (fluvial) flooding is the most common flood disaster globally, the majority of research incorporating mental health impact has focused on floods that arise from typhoons/cyclones and coastal surges (extreme tides combined with severe storms) (10). Fluvial flooding has unique characteristics in that it occurs after extended periods of heavy rain within river catchments that can lead to high velocity, large volume coastal, and inland flows with little warning. Within the context of increasing frequency of extreme rainfall events (due to a warming climate and intensified hydrological cycles) and urbanization of flood zones, the probability of flood events occurring and their intensity will further increase the severity of human impacts (11). One such event occurred in late March/early April 2017, with heavy rainfall from ex-Tropical Cyclone Debbie (the second most destructive cyclone in Australia) (12) causing devastating flooding in Queensland, Northern New South Wales (NSW) and subsequently the North Island of New Zealand. Record breaking rainfall occurred in Northern NSW (12). In Lismore (one of the larger population centers in the region with over 25,000 residents) (13), the levee was overtopped for the first time and the ensuing flood was the worst since 1974, inundating the central business district and low-lying residential areas close to town (14). Murwillumbah (population ~9,000) in the Tweed River Valley experienced its highest flood level in recorded history (14).

In Australia, inequality in the distribution of income and wealth has resulted in sectors of the community experiencing significant poverty, disproportionately so within the Aboriginal and Torres Strait Islander population (15, 16). Compared to NSW overall, the Northern NSW region has: higher proportions of people living with underlying vulnerability; lower median household incomes; and greater government income support reliance (e.g., single parent, disability, unemployment, and youth payments) (13). The region also has a higher proportion of Aboriginal people (4.1%) compared to the state average (2.9%) (13). The region experiences fluvial flooding regularly (over 30 flood disaster declarations in the decade 2004–2014) (17), yet there is little information about underlying risk, that is, individual, and community-level factors that mediate flood impact on mental health which, in turn influences the adaptive capacity of the community to climate change effects.

As espoused by the Sendai Framework, this project aims to understand the interplay of factors that may contribute to local disaster risk and adaptive capacity to inform risk reduction policies. We utilized a systems thinking-based social-ecological approach in a community-academic partnership to develop a “*flood impact on mental health framework”* (the framework) (18). It describes putative relationships between flood exposure and mental health and well-being and maps the influence of mediating factors from personal (e.g., socio-demographic factors, “personal social capital,” and individual social support), community (e.g., community cohesion) and organizational levels of analysis (such as pre-flood mitigation systems, disaster relief responses, and community and health service responses) (18). Our objectives were to explore the relationships described within the framework with a focus on key interest groups within our region, such as farmers, business owners, young adults (16–25 years), older people aged over 75 years and socio-economically marginalized subpopulations (18). The project forms the baseline for a planned longitudinal cohort study to improve understanding of mental health and well-being impact from river flooding in the short (1–2 years) and medium-term (3–5 years). It will enable identification of opportunities to mitigate risk and inform strategies to strengthen public health services and psychosocial resilience to future flooding.

This paper presents initial results from the project and contributes new knowledge by quantifying the associations between intensity of fluvial flood exposure (how many sites of importance were flooded) and five mental health problems. These include two directly event-linked problems [still distressed about the flood and flood-related post-traumatic stress disorder (PTSD)] and illustrates how these associations vary according to socio-economic circumstance. There is also opportunity to contribute learnings to an international initiative tracking health impacts from climate change (19).

## Methods

### Study Design

**Figure 1:**
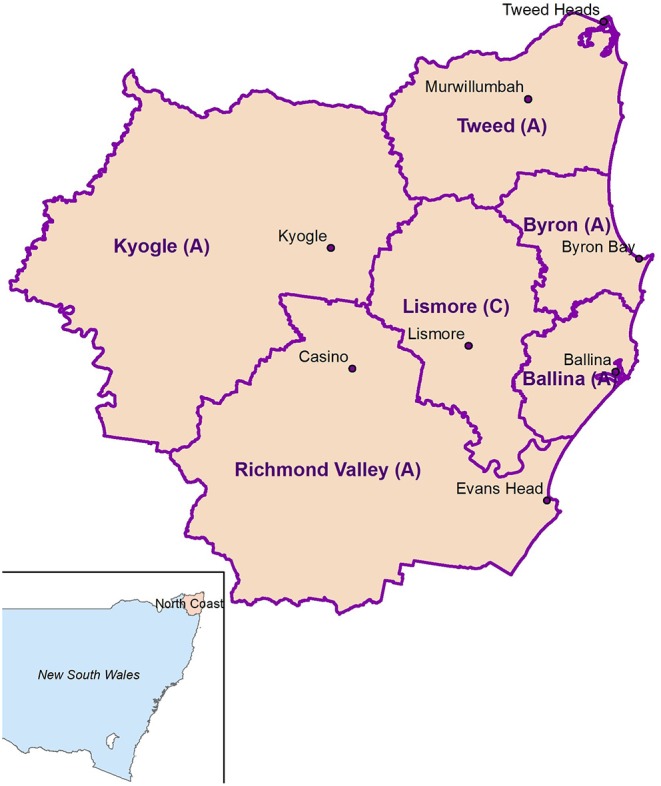
Study location.

A cross-sectional survey was undertaken 6 months after flooding in communities within six Northern NSW Local Government Areas (Ballina Shire, Tweed Shire, Richmond Valley, Kyogle, Byron Shire and Lismore City) (Figure 1) which have an estimated population of 247,000 (~202,000 aged 15 years and over) (13). Community members 16 years and older resident in Northern NSW at the time of the flood were invited to participate.

The project's community-academic partnership approach was integral to the design, recruitment to and implementation of the study (18). It included the recruitment of local community members to promote the survey and the establishment of two project specific Community Advisory Groups (CAGs) in the regional centers, Lismore and Murwillumbah. The CAGs comprised local health and community organizations, business groups, and state and local government authorities who have responsibility for flood planning, emergency response, mental health service provision, and/or advocacy and support for the project's key interest groups. Guided by the framework and together with experts in survey design and in floods and mental health research, the CAGs reviewed the face and content validity of the questionnaire and proposed topics that should be included (e.g., level and perceived adequacy of support received from government and community agencies at the time of the flood). The survey was piloted with 30 individuals from various socio-demographic backgrounds (recruited via the CAGs and the research team) and subsequently revised.

The survey was available online and in paper form between September and November 2017. The online version of the survey, suitable for use on computers and mobile telephones, was generated using Qualtrics software (version Sept–Nov 2017, Qualtrics Provo Utah). Potential respondents were provided with participant information and advised that completion of the questionnaire would signify consent to participate in the study.

We utilized a purposive snowball sampling technique to recruit respondents via personal, social and local organizational networks, the CAGs and other business groups and community organizations. This was supplemented by an extensive local media campaign (print, broadcast, and social media), advertising campaign and a door-to-door survey conducted at the end of the recruitment period in randomly selected neighborhood blocks from flooded areas of Lismore and Murwillumbah (to assess response bias, participation rates, and effectiveness of recruitment strategies) (18). The advertising campaign included posters and paper surveys (with reply-paid postage) left in central community locations such as post offices, libraries, coffee shops and store fronts of charitable organizations such as Lifeline, St Vincent de Paul and the Salvation Army. Project staff promoted the survey at various community events including farmers' markets, and through the local postal service, we deposited postcards in residential mailboxes with information on accessing the survey. As an incentive, we also offered respondents an opportunity to enter a lottery style draw upon survey completion for a $100 local shopping voucher (18).

Our aim was to recruit participants from the local community experiencing different degrees of flood impact, including the key groups described earlier, some of which are hard to reach via conventional sampling strategies. Where certain subgroups are few in number and a degree of trust is required to support their participation, community-partnered snowball sampling approaches or “ascending” methods (working from the ground up) are preferable to “descending” methods, such as household surveys (20, 21). As our interest was to quantify relationships between flood impact and psychological morbidity, extrapolation to other populations was a secondary consideration (22). Our sampling methodology therefore targeted our key interest groups as well as a broad cross-section of the community, encouraging residents to participate regardless of whether they felt the 2017 flood had affected them.

### Measures

The questionnaire included participants' socio-demographic characteristics, flood exposure measures (including evacuation and displacement) and mental health screening tools. Socio-demographic data were age, sex, Aboriginal, and Torres Strait Islander status, relationship status, employment status, type of income support payments, and educational qualifications.

Flood exposure measures were selected *a priori* and included self-reported damage to five physical infrastructure sites: suburb; non-liveable areas of their home (e.g., garden shed, garage); liveable areas of their home (e.g., bedrooms); income-producing property (business/farm); and the home of a significant other as well as evacuation and length of displacement. Those who did not indicate any of these exposures were termed “non-exposed” and this group formed the internal comparison against which exposed groups were compared. To examine cumulative impacts, we derived a cumulative exposure index for individuals by summing the number of damage sites experienced. The index ranged from zero (no sites damaged) to five (experienced all five).

We measured health status using brief clinical screening tools for depression, anxiety, and post-traumatic stress. The Patient Health Questionnaire (PHQ-2) for depression has previously shown acceptable diagnostic accuracy, reliability, construct and criterion validity, and sensitivity to change in primary care and other clinic settings in western countries (23, 24). The Generalized Anxiety Disorder scale (GAD-2) has shown acceptable diagnostic accuracy from meta-analysis of validation studies in western countries (pooled sensitivity 0.76 and specificity 0.81) (25, 26). The Post Traumatic Stress Disorder Checklist (PCL-6) has shown adequate diagnostic performance in United States primary care settings (sensitivity 0.80 and specificity 0.76) including for underserved and minority populations (27, 28). Cut-points for probable diagnosis were ≥3 for the PHQ-2/GAD-2 and ≥14 for the PCL-6 (23, 25, 27). For the PCL-6, the checklist was introduced as a list of complaints that people sometimes have after severe rain and flooding to relate responses to the flood. We also used a single suicidal ideation item from the Screening Tool for Assessing Risk of Suicide (“Over the past 4 weeks, have you personally had any thoughts about ending your life?”) (29) and a single measure of continuing impact 6 months after the flood (“Are you still currently distressed about what happened during the flood?”) from the Brief Weather Disaster Trauma Exposure and Impact Screen (30).

### Participants

A total of 2,530 people responded to the survey (76% online), 350 (14%) of whom were excluded from the primary analyses because of missing socio-demographic data, leaving a final sample of 2,180. Minimal differences in parameter estimates and no differences in patterns of results were found between the full dataset and the dataset with missing socio-demographic records removed (Supplementary Table 1). Respondents were predominantly women (69%) and people aged between 35 and 74 years (82%). ~4% of respondents were Aboriginal and/or Torres Strait Islander and over a quarter of respondents were receiving income support at the time of the flood (Table 1). Recruitment strategies were successful in raising awareness of the survey with around 50% of residents within the door-knock areas (18). Of those door-knocked, ~5% had already completed the survey, the majority of which were women (69%). The sampling strategy was not intended to obtain representation of the broader Northern NSW population, but rather to obtain respondents in each category of interest to enable comparison of experience among the key interest groups.

**Table 1:** Socio-demographic profile of survey respondents by exposure category.

			**Flood exposure damage** ***n*** **(%)**					
		***n* (%)**	**Non-exposed**	**Home of a significant other**	**Suburb**	**Non-liveable areas of home**	**Liveable areas of home**	**Business/farm**
Total respondents		2,180 (100)	198 (9)	1,380 (63)	1,659 (76)	1,035 (47)	460 (21)	365 (17)
Age group	16–34	309 (14)	19 (10)	215 (16)	234 (14)	141 (14)	68 (15)	35 (10)
	35–54	902 (41)	79 (40)	593 (43)	687 (41)	435 (42)	192 (42)	173 (47)
	55–74	894 (41)	85 (43)	542 (39)	685 (41)	433 (42)	188 (41)	150 (41)
	75+ years	75 (3)	15 (8)	30 (2)	53 (3)	26 (3)	12 (3)	7 (2)
Gender	Women	1,500 (69)	128 (65)	963 (70)	1144 (69)	713 (69)	309 (67)	225 (62)
	Men	680 (31)	70 (35)	417 (30)	515 (31)	322 (31)	151 (33)	140 (38)
Indigenous status	Indigenous	77 (4)	3 (2)	67 (5)	58 (3)	50 (5)	28 (6)	9 (2)
	Non-indigenous	2,103 (96)	195 (98)	1313 (95)	1601 (97)	985 (95)	432 (94)	356 (98)
Relationship status	Single	704 (32)	49 (25)	469 (34)	556 (34)	374 (36)	188 (41)	73 (20)
	In a relationship/ married	1,476 (68)	149 (75)	911 (66)	1103 (66)	661 (64)	272 (59)	292 (80)
Education level	University degree	957 (44)	100 (51)	576 (42)	701 (42)	405 (39)	162 (35)	146 (40)
	Other	1,223 (56)	98 (49)	804 (58)	958 (58)	630 (61)	298 (65)	219 (60)
Employment status	Paid employment (part- or full-time)	1,511 (69)	125 (63)	967 (70)	1140 (69)	681 (66)	278 (60)	298 (82)
	Other	669 (31)	73 (37)	413 (30)	519 (31)	354 (34)	182 (40)	67 (18)
Income support^*^	Yes	643 (29)	53 (27)	434 (31)	523 (32)	376 (36)	195 (42)	60 (16)
	No	1,537 (71)	145 (73)	946 (69)	1,136 (68)	659 (64)	265 (58)	305 (84)

* *Income support at time of the flood: age pension; veteran payment; single parent support; unemployment support; youth allowance; education support; disability support pension; carer payment*.

### Statistical Methods

Separate binary logistic regression models were constructed to calculate the odds of experiencing symptoms (yes/no) related to five types of mental health problem (continuing distress, suicidal ideation or probable depression, anxiety, or PTSD) by single exposure (damage to suburb, non-liveable areas, liveable areas, and home of a significant other and evacuation and length of displacement) as well as cumulative flood exposure relative to the non-exposed group. We adjusted the models for all measured socio-demographic characteristics. Potential interactions between these characteristics were checked for significance. Respondents who did not complete a health outcome measure were excluded from analysis for that indicator only. Adjusted predictions of the probability of reporting a mental health outcome for different levels of exposure were calculated by using the marginal standardization method. As we conducted multiple analyses, the *p*-value was set conservatively at <0.01. Stata (version 15, StataCorp) was used for statistical analysis.

## Results

About nine-in-ten respondents reported being affected in some way while ~9% were classified as non-exposed (no damage to surrounding infrastructure, no evacuation or displacement). Around three-quarters reported suburb damage and almost two-thirds had a home of a significant other flooded (Table 1). Liveable areas were flooded in the homes of over one-in-five respondents while almost as many had their income-producing properties (businesses/farms) flooded. Compared to their proportions in the total respondent group, there were higher proportions of Aboriginal and Torres Strait Islander people (6 vs. 4%), single people (41 vs. 32%), those not in paid employment (40 vs. 31%) and income support recipients (42 vs. 29%) who reported flooding in liveable areas of their home. Approximately 14% (*n* = 315) of respondents reported being displaced and 4% (*n* = 85) were still living elsewhere 6 months after the flood (Table 2).

**Table 2:** Adjusted odds ratios (AOR) of mental health outcomes across exposure measures compared with “non-exposed” respondents (*N* = 2,180).

			**Still distressed (*****n*** **=** **486; 22%)**			**Probable PTSD (*****n*** **=** **332; 15%)**			**Probable anxiety (*****n*** **=** **343; 16%)**			**Probable depression (*****n*** **=** **335; 15%)**			**Suicidal ideation (*****n*** **=** **159; 7%)**		
Exposure***^#^***		***N***	***n* (%)**	**AOR^∧^**	**99% CI**	***n* (%)**	**AOR^∧^**	**99% CI**	***n* (%)**	**AOR^∧^**	**99% CI**	***n* (%)**	**AOR^∧^**	**99% CI**	***n* (%)**	**AOR^∧^**	**99% CI**
Non-exposed		198	12 (6)	1.00		6 (3)	1.00		11 (6)	1.00		13 (7)	1.00		10 (5)	1.00	
Home of significant other affected		1380	380 (27)	5.53	(2.51–12.20)^**^	259 (19)	7.36	(2.24–24.19)^**^	260 (19)	3.21	(1.40–7.38)^**^	247 (18)	2.80	(1.24–6.30)^*^	111 (8)	1.37	(0.56–3.36)
Suburb affected		1659	440 (27)	5.09	(2.32–11.18)^**^	306 (18)	6.09	(2.04–18.13)^**^	303 (18)	3.14	(1.37–7.19)^**^	291 (18)	2.48	(1.13–5.43)^*^	141 (9)	1.46	(0.60–3.56)
Non-liveable areas affected		1035	347 (34)	7.00	(3.17–15.50)^**^	247 (24)	8.32	(2.78–24.86)^**^	228 (22)	3.92	(1.70–9.05)^**^	220 (21)	3.06	(1.39–6.75)^**^	109 (11)	1.75	(0.71–4.32)
Liveable areas affected		460	217 (47)	12.14	(5.36–27.47)^**^	161 (35)	13.72	(4.53–41.56)^**^	137 (30)	5.42	(2.29–12.79)^**^	134 (29)	4.37	(1.93–9.89)^**^	68 (15)	2.59	(1.02–6.62)^*^
Evacuated home		333	151 (45)	9.87	(4.25–22.92)^**^	118 (35)	14.53	(4.29–49.24)^**^	106 (32)	5.40	(2.23–13.06)^**^	97 (29)	4.21	(1.76–10.08)^**^	52 (16)	2.58	(0.97–6.88)
Displaced <6 months		230	75 (33)	6.34	(2.65–15.13)^**^	64 (28)	9.73	(3.08–30.78)^**^	53 (23)	4.17	(1.66–10.48)^**^	49 (21)	2.98	(1.22–7.26)^*^	34 (15)	2.46	(0.90–6.75)
Displaced ≥ 6 months		85	57 (67)	25.70	(9.20–71.81)^**^	46 (54)	24.43	(7.05–84.69)^**^	45 (53)	14.50	(5.15–40.85)^**^	38 (45)	8.38	(3.04–23.10)^**^	18 (21)	3.17	(0.96–10.39)
Business/farm affected		365	134 (37)	8.36	(3.62–19.28)^**^	89 (24)	11.60	(3.63–37.07)^**^	88 (24)	5.47	(2.24–13.40)^**^	81 (22)	4.28	(1.81–10.13)^**^	43 (12)	2.88	(1.06–7.85)^*^
Evacuated business		305	114 (37)	8.79	(3.68–20.99)^**^	72 (24)	13.59	(3.90–47.40)^**^	73 (24)	5.57	(2.17–14.30)^**^	71 (23)	5.00	(2.00–12.55)^**^	36 (12)	2.94	(1.03–8.40)^*^
Cumulative exposure index^†^	1	428	34 (8)	1.30	(0.53–3.21)	18 (4)	1.29	(0.37–4.48)	29 (7)	1.10	(0.43–2.86)	34 (8)	1.11	(0.45–2.72)	19 (4)	0.79	(0.28–2.26)
	2	551	84 (15)	2.82	(1.22–6.47)^*^	54 (10)	3.20	(1.02–10.01)^*^	74 (13)	2.39	(1.00–5.73)	67 (12)	1.79	(0.77–4.13)	18 (3)	0.56	(0.19–1.61)
	3	514	136 (26)	5.30	(2.34–11.98)^**^	99 (19)	6.43	(2.11–19.60)^**^	86 (17)	2.77	(1.17–6.61)^*^	91 (18)	2.48	(1.09–5.65)^*^	49 (10)	1.74	(0.68–4.46)
	4	383	177 (46)	12.68	(5.57–28.85)^**^	121 (32)	11.61	(3.80–35.49)^**^	109 (28)	5.24	(2.20–12.46)^**^	103 (27)	3.93	(1.72–8.98)^**^	45 (12)	1.93	(0.74–5.03)
	5	59	40 (68)	31.85	(10.99–92.27)^**^	31 (53)	32.80	(9.07–118.63)^**^	29 (49)	14.63	(5.02–42.64)^**^	24 (41)	8.41	(2.91–24.34)^**^	18 (31)	7.87	(2.48–25.05)^**^

*CI, confidence interval; N, number within the total sample experiencing the exposure; n, number within the exposure category with the mental health outcome measure*.

# *Exposure categories are not mutually exclusive, hence comparison across exposures must be treated with caution particularly if there are marginal differences between estimates*.

∧ *Adjusted for age, sex, Aboriginal and Torres Strait Islander status, relationship status, education qualification, employment status, and income support*.

* p < 0.01;

** *p < 0.001*.

† *Cumulative exposure index is the sum of exposures experienced: home of a significant other + suburb + non-liveable area of home + liveable area of home + business/farm. It ranges from zero (non-exposed) to five (all five exposures). For unadjusted analyses, please see Supplementary Table 1*.

Over one-fifth (22%) of respondents reported being still distressed about the flood, 16% with probable anxiety, 15% probable PTSD, 15% probable depression and 7% suicidal ideation. Around 27% of respondents reported at least one of these and about 20% reported two or more of these problems. The odds of any mental health problem were significantly elevated across most exposure measures compared with the non-exposed group, particularly those whose homes and/or businesses/farms were evacuated or flooded and those who were still displaced after 6 months (Table 2). Respondents who had their homes or businesses inundated had between two to three times greater odds of reporting suicidal ideation than the non-exposed group.

Increasing intensity of exposure, as indicated by the cumulative exposure index, was associated with the likelihood of progressively worse mental health (Table 2). For example, for every incremental increase in the index, there was an exponential doubling of the odds across exposure levels of continuing distress and PTSD compared to non-exposed respondents. The predicted probability of reporting continuing distress for someone scoring one on the index was 8% and it was 67% for someone scoring five (5 and 52%, respectively for PTSD) (Table 3).

**Table 3:** Predicted probability (% & 99%CIs) of reporting mental health problems by number of exposures and length of displacement (less than or more than 6 months).

			**Still distressed**		**PTSD**		**Anxiety**		**Depression**		**Suicidal ideation**	
		***N* (%)**	**%**	**99% CIs**	**%**	**99% CIs**	**%**	**99% CIs**	**%**	**99% CIs**	**%**	**99% CIs**
Cumulative exposure index	1	428 (20)	8	(5–12)	5	(2–7)	7	(4–11)	9	(5–13)	5	(2–7)
	2	551 (25)	16	(12–20)	11	(7–17)	15	(11–18)	13	(10–17)	3	(1–5)
	3	514 (24)	26	(21–31)	19	(14–23)	16	(12–21)	17	(13–21)	10	(6–13)
	4	383 (18)	46	(39–52)	29	(23–35)	26	(21–32)	24	(19–29)	10	(7–14)
	5	59 (3)	67	(52–83)	52	(35–68)	49	(32–65)	39	(23–54)	30	(15–45)
Displacement (months)	<6	230 (11)	32	(24–40)	27	(20–34)	23	(16–30)	20	(14–27)	14	(8–20)
	≥6	85 (4)	64	(50–78)	47	(33–62)	49	(34–64)	39	(26–53)	17	(7–27)

Of those displaced, 58% had their homes flooded. Other evacuees whose homes were not flooded lived elsewhere for other reasons including damaged roads and landslips. Out of the 230 people who returned home within 6 months, 56% (*n* = 129) did so within 4 days. Compared to short-term evacuees, those displaced for longer than 6 months were twice as likely to report being still distressed and having symptoms of PTSD, anxiety and depression (Table 3).

The results of the logistic regression analyses for Aboriginal and Torres Strait Islander respondents and respondents in receipt of income support are presented in Table 4. Compared to others (and based on unadjusted odds ratios), these respondents were more likely to be evacuated, have their homes inundated and/or be displaced for 6 months or more. While there was a higher proportion of Aboriginal and Torres Strait Islander respondents receiving income support payments (44%) compared to non-Indigenous respondents (30%), there was no significant interaction between these socio-demographic categories with respect to reporting flood exposures or mental health outcomes. After adjusting for severity of flood exposure (cumulative exposure index), Aboriginal and Torres Strait Islander respondents were significantly more likely to report probable anxiety and depression and income support recipients were more likely to report probable PTSD, anxiety, depression, and suicidal ideation compared to other respondents (Table 4).

**Table 4:** Odds ratios of flood exposure and mental health outcomes for Aboriginal and Torres Strait Islander respondents and respondents in receipt of income support (*N* = 2,180).

**Respondents who reported being**		**Aboriginal & Torres Strait Islander (*****n*** **=** **77)**		**In receipt of income support (*****n*** **=** **643)**	
		**(Reference** **=** **non-Indigenous)**		**(Reference** **=** **no income support)**	
		**Odds ratio^#^**	**99% CI**	**Odds ratio^#^**	**99% CI**
Sites of flood damage	Home of significant other	4.35	(1.73–10.93)^**^	1.35	(1.04–1.75)^*^
	Suburb damaged	0.95	(0.48–1.90)	1.53	(1.13–2.06)^**^
	Non-liveable area	2.05	(1.10–3.84)^*^	1.91	(1.49–2.45)^**^
	Liveable area	2.28	(1.21–4.29)^*^	2.16	(1.63–2.87)^**^
	Home evacuation	2.87	(1.50–5.50)^**^	2.29	(1.68–3.14)^**^
	Had to live elsewhere	2.00	(0.99–4.03)	2.13	(1.55–2.94)^**^
	Displaced ≥6months	3.04	(1.11–8.33)^*^	3.81	(2.13–6.84)^**^
	Business/farm damaged	1.02	(0.49–2.10)	1.04	(0.77–1.39)
		**Adjusted odds ratio**^∧^	**99% CI**	**Adjusted odds ratio**^∧^	**99% CI**
Mental health outcomes	Still distressed	1.93	(0.96–3.86)	1.34	(0.92–1.97)
	Probable PTSD	1.88	(0.91–3.88)	1.75	(1.15–2.68)^*^
	Probable anxiety	2.16	(1.08–4.33)^*^	1.89	(1.26–2.85)^**^
	Probable depression	2.09	(1.04–4.23)^*^	1.84	(1.22–2.79)^**^
	Suicidal ideation	0.67	(0.22–2.04)	1.85	(1.06–3.25)^*^

# *Unadjusted odds ratio*.

∧ *Adjusted odds ratio for other socio-demographic variables and severity of flood exposure*.

* p < 0.01;

** *p < 0.001*.

## Discussion

Our results demonstrate elevated psychological morbidity among survey respondents 6 months after the 2017 severe flooding in Northern NSW with greater impact on marginalized respondent groups. Rates of still being distressed about the flood, probable PTSD, anxiety, and depression, and suicidal ideation were particularly elevated in response to three types of exposure: those whose homes or businesses were flooded; those who faced multiple exposures; and those who endured lengthy displacement. Respondents already experiencing socio-economic marginalization were more likely to be exposed and, if exposed, to have elevated risk of psychological morbidity (i.e., after accounting for extent of flood damage).

Our findings are in keeping with those of previous studies describing how flooding across different scenarios of impact can harm mental health (30–32). For instance, after severe cyclones buffeted Queensland in the summer of 2010–11, flood damage to areas outside individuals' homes (e.g., in their suburbs, the homes of close relatives/friends, and income-producing properties) was linked to elevated rates of mental health problems, and residents in the most disadvantaged areas were more likely to report home damage. Further, if exposed to these forms of damage, they were likely to report much higher rates of psychiatric morbidity than equally-exposed people in more advantaged areas (30). We add to this knowledge in five ways: by discriminating between damage inside and immediately outside the home (yards/gardens); by quantifying the associations between intensity of exposure (how many of five different places were flooded) and psychological impact; by investigating impacts across five mental health problems, including two that were directly event-linked (still distressed about the flood and flood-related PTSD); by quantifying the nature and amplified degree of impacts on specific marginalized sub-population groups; and by examining fluvial flooding impacts in a rural area of New South Wales.

There was an exponential increase in the likelihood of respondents experiencing continuing distress and flood-related PTSD with each additional exposure. For example, while there was no substantial difference in mental health outcomes between respondents experiencing one exposure compared to non-exposed, those reporting three exposure sites (e.g., home and business and suburb) had, respectively, five and six times the odds of reporting continuing distress and PTSD. Further, while immediate-term evacuation and displacement are known stressors (6, 32), our findings suggest that lengthy displacement is associated with particularly high levels of mental health risk: respondents “still not home” after 6 months had double the probability of reporting continuing distress and symptoms of PTSD, anxiety, and depression when compared to those who were briefly displaced. With almost one-half of respondents reporting three or more exposures and a small but important minority displaced long-term (most of whom also experienced multiple exposures), there is a sub-group of people with a high-risk profile for significant psychological burden following the flood. These people have extensive immediate and medium-term social and health needs and are at elevated risk of long-term psychological morbidity (32). Further investigation into issues prolonging displacement, such as lack of financial assistance and onerous insurance processes (33) as well as research into the causes and effects of multiple domains of exposure and impact, are required to fully understand how these factors interact to shape mental health, and to minimize risk and build resilience.

In our study, Aboriginal and Torres Strait Islander respondents and respondents in receipt of income support fall disproportionately within the high-risk sub-group described above. It is recognized that elevated risk of psychological morbidity is pre-existing for these groups due to their poorer underlying health and socio-economic status (6). This double disadvantage is a significant issue in characterizing the potential impacts of climate change (8). For example, compared to non-Indigenous respondents, Aboriginal and Torres Strait Islander respondents had four times the odds of reporting damage to the home of a significant other. Extended close family and community connections may form a protective factor for Aboriginal and Torres Strait Islander communities when faced with adversity (34) but this very closeness may also be a risk factor. That is, the more closely connected a community is, the more it may be likely to “feel” harm to its members. This may help explain why Aboriginal and Torres Strait Islander people are at high risk of disruption to mental health-protective close social connections and support when their communities experience a flood disaster. Understanding the contexts operating within subpopulations will help inform intervention strategies that build on existing strengths to promote resilience and pre-emptively address key vulnerabilities.

### Implications for Public Health

Our findings have improved understanding of the local context by highlighting the relationship between severity of flood exposure and mental health outcomes, including for respondents most in need. Joint design and analysis of the study with community representatives has enabled the sharing of knowledge and recognition of strengths and gaps in local policy and practice, particularly for at-risk groups. For example, NSW emergency services engage with non-government welfare agencies to provide immediate post-disaster support. Our findings underscore the importance of these initiatives and indicate the additional necessity for first responders to be able to assess and react appropriately to multiple or high-risk exposures. Care pathways that are individually tailored and sensitive to specific exposures and risk factors may be more effective in preventing the onset of symptoms and in promoting recovery. In addition, the focus of disaster recovery programs needs to be extended beyond the immediate aftermath given research has shown that mental health problems persist for many years (35). Anecdotal evidence from local service providers in Northern NSW indicated low uptake of mental health services established immediately after the flood. Our next stage of research will focus on the changing nature of mental health needs of respondents over time following a disaster.

More generally, a multi-sectoral agency approach in disaster preparedness and response, consistent with the guidelines from the Sendai Framework, should be used to promote flexible services adapted to meet the needs of community members according to their economic and social circumstance (8). For health systems, this includes empowering people through inclusive processes in designing strategies to mitigate their risks before, during and after disasters, especially among those who may be disproportionately affected by disasters (8). Systems-level focus and action is required to move beyond individual behavioral change interventions (where success relies on individual capacity, opportunity, and resources) toward group-level change strategies that can involve everyone regardless of circumstance and build communities' social capital and underlying resilience (5). Community development approaches, in which local government and community services collaborate to promote social cohesion and well-being, have proven effective in moderating the mental health impacts of persistent drought in rural NSW (36). With guidance from the project's Community Advisory Groups, similar approaches could work for flood-prone communities in the Northern NSW region.

### Strengths and Limitations

Our capacity to generalize our findings to other settings has limitations. This is a self-report, cross-sectional design which constrains our ability to make causal inferences: pre-existing mental health status can bias responses and we cannot be sure flood experiences directly caused outcomes (5). Further, our sampling approach was not intended to and should not be used to estimate population prevalence for either exposure or for outcome measures. We recommend the magnitude of the adjusted odds ratios and associated confidence intervals reported in our study be interpreted in relation to the sampling approach of our survey and that the risk estimates of psychological outcomes between specific subgroups be interpreted with caution. Nevertheless, our findings are consistent with and meaningfully extend the findings of previous studies which employed potentially more robust (and costly) conventional survey techniques, such as random-digit dialing (landline telephones) and household mailouts (30, 31). Indeed, these studies often report low response rates, selection bias, difficulty identifying appropriate sampling frames and delays in capturing post-event data. They also recognize their inability to adequately capture the experiences of displaced populations. Our pragmatic, purposive sampling approach was able to overcome some of these limitations, enabling us to measure disaster experiences within diverse and hard-to-reach sub-population groups.

We included two mental health measures specifically related to the flood (including PTSD) as well as general measures of depression, anxiety, and suicidal ideation, and we adjusted our analyses by a wide variety of socio-economic factors known to predict psychological morbidity (37). A particular strength of our study [consistent with recommendations from the Sendai Framework (8)] was the inclusion of multiple dimensions of exposure and vulnerability to describe disaster risk. We achieved this by engaging closely with the community from the outset, utilizing local community, and organizational networks to document experiences of socio-economically marginalized respondents. This co-production and evaluation of knowledge means that our findings are able to directly address community identified priorities. This means, in turn, that our findings are relevant to local organizations' and governments' role in strengthening public health policy and service development processes related to climate change and associated extreme weather events.

## Conclusion

Six months after the 2017 Northern NSW flood event, survey respondents revealed a substantial continuing mental health burden; we have characterized and quantified this burden and its inequitable distribution in a rural Australian context. The community-academic partnership approach used in this study means that local communities helped generate the knowledge they need to begin work to address the findings. In the context of climate change, weather disasters will become increasingly frequent, intense, and unpredictable with the potential for correspondingly severe effects on mental health. A recent systems framework highlights the complexity of interactions between climate change and mental health (5). Such frameworks encourage research partnerships to trial tailored adaptation interventions aimed at building community cohesion and disrupting the pathways of harm that link climate change and mental health. Our study is an early example of such an approach. We have an opportunity to establish long-term collaborative research to develop and evaluate such interventions. These further studies will help describe the scale, intensity, and duration of climate change related mental health impacts in a rural setting, assist with stakeholder driven assessment and strengthening of mental health support systems and, therefore, help formulate effective adaptation for an Australian community most vulnerable to extreme weather events.

## Data Availability Statement

The datasets generated for this study are available on request to the corresponding author.

## Ethics Statement

The study was approved by the University of Sydney Human Research Ethics Committee (reference-2017/589) and the Aboriginal Health and Medical Research Council Human Research Ethics Committee (reference-1294/17). Written informed consent from the participants' legal guardian/next of kin was not required to participate in this study in accordance with the national legislation and the institutional requirements.

## Author Contributions

VM performed the data analysis. VM, JL, and HB interpreted the data and drafted the manuscript. All authors contributed to the study design, critically reviewed draft versions and provided important intellectual content during revisions, and accept accountability for the overall work.

### Conflict of Interest

The authors declare that the research was conducted in the absence of any commercial or financial relationships that could be construed as a potential conflict of interest.
