# Supplementary material for: Differential Mental Health Impact Six Months After Extensive River Flooding in Rural Australia: A Cross-Sectional Analysis Through an Equity Lens
Source: Front Public Health. 2019 Dec 6;7:367. doi: 10.3389/fpubh.2019.00367 (PMC6909816; doi:10.3389/fpubh.2019.00367)
Supplement: Supplementary file 1 [file Table_1.docx]

Supplementary Table 1 Results of sensitivity analysis showing unadjusted odds ratio (UOR) and 99% CIs of mental health impact from different exposures for all data (n=2,530) and for dataset where missing socio-demographic information has been removed (n=2,180). (CI=confidence interval; n=number within the exposure category with the mental health outcome measure; ^†^Cumulative exposure index is the sum of exposures experienced: home of a significant other + suburb + non-liveable area of home + liveable area of home + business/farm. It ranges from zero (non-exposed) to five (all five exposures); ^*^p<0.01; ^**^p<0.001).

|  |  | **Still distressed** | | | | | | | | **Probable PTSD** | | | | | | | | |
| --- | --- | --- | --- | --- | --- | --- | --- | --- | --- | --- | --- | --- | --- | --- | --- | --- | --- | --- |
|  |  | **All records** | | | | **Records with missing socio-demographic data removed** | | | | | **All records** | | | | **Records with missing socio-demographic data removed** | | | |
|  |  | **n** | **UOR** | **99%CI** |  | **n** | **UOR** | **99%CI** |  | | **n** | **UOR** | **99%CI** |  | **n** | **UOR** | **99%CI** |  |
| **Home of significant other damaged** |  | 418 | 5.57 | (2.70-11.52) | ^**^ | 380 | 5.77 | (2.63-12.63) | ^**^ | | 281 | 9.25 | (2.85-30.07) | ^**^ | 259 | 8.58 | (2.63-27.95) | ^**^ |
| **Suburb damaged** |  | 491 | 5.35 | (2.60-11.04) | ^**^ | 440 | 5.54 | (2.53-12.09) | ^**^ | | 337 | 7.60 | (2.58-22.34) | ^**^ | 306 | 7.06 | (2.39-20.80) | ^**^ |
| **Non-liveable areas damaged** |  | 388 | 7.65 | (3.69-15.86) | ^**^ | 347 | 7.78 | (3.54-17.08) | ^**^ | | 271 | 10.55 | (3.57-31.14) | ^**^ | 247 | 9.76 | (3.30-28.89) | ^**^ |
| **Liveable areas damaged** |  | 244 | 13.42 | (6.36-28.30) | ^**^ | 217 | 13.93 | (6.23-31.16) | ^**^ | | 173 | 17.13 | (5.73-51.16) | ^**^ | 161 | 16.68 | (5.56-50.01) | ^**^ |
| **Evacuated home** |  | 168 | 12.61 | (5.89-26.98) | ^**^ | 151 | 13.02 | (5.73-29.57) | ^**^ | | 124 | 20.09 | (6.05-66.74) | ^**^ | 118 | 20.15 | (6.04-67.21) | ^**^ |
| **Displaced <6 months** |  | 88 | 7.80 | (3.55-17.14) | ^**^ | 75 | 7.64 | (3.27-17.89) | ^**^ | | 70 | 12.35 | (4.00-38.10) | ^**^ | 64 | 11.97 | (3.85-37.20) | ^**^ |
| **Displaced ≥6 months** |  | 60 | 30.14 | (12.07-75.26) | ^**^ | 57 | 31.84 | (12.00-84.47) | ^**^ | | 46 | 35.48 | (10.70-117.69) | ^**^ | 46 | 36.17 | (10.82-120.90) | ^**^ |
| **Business/farm damaged** |  | 149 | 8.48 | (3.97-18.11) | ^**^ | 134 | 8.84 | (3.90-20.04) | ^**^ | | 99 | 10.74 | (3.55-32.55) | ^**^ | 89 | 10.02 | (3.29-30.53) | ^**^ |
| **Evacuated business** |  | 125 | 8.45 | (3.92-18.19) | ^**^ | 114 | 9.24 | (4.04-21.13) | ^**^ | | 82 | 10.67 | (3.49-32.57) | ^**^ | 72 | 9.77 | (3.17-30.06) | ^**^ |
| **Cumulative exposure index^†^** | **1** | 39 | 1.25 | (0.55-2.89) |  | 34 | 1.31 | (0.54-3.22) |  | | 19 | 1.41 | (0.41-4.79) |  | 18 | 1.39 | (0.40-4.77) |  |
|  | **2** | 96 | 2.73 | (1.27-5.87) | ^*^ | 84 | 2.75 | (1.21-6.29) | ^*^ | | 63 | 3.89 | (1.27-11.94) | ^*^ | 54 | 3.39 | (1.10-10.51) | ^*^ |
|  | **3** | 153 | 5.53 | (2.61-11.72) | ^**^ | 136 | 5.50 | (2.45-12.36) | ^**^ | | 108 | 7.98 | (2.65-24.02) | ^**^ | 99 | 7.42 | (2.45-22.45) | ^**^ |
|  | **4** | 190 | 12.78 | (6.01-27.20) | ^**^ | 177 | 13.48 | (5.98-30.39) | ^**^ | | 128 | 15.27 | (5.07-45.98) | ^**^ | 121 | 14.44 | (4.78-43.62) | ^**^ |
|  | **5** | 48 | 28.94 | (11.15-75.08) | ^**^ | 40 | 31.75 | (11.10-90.84) | ^**^ | | 36 | 34.11 | (9.97-116.72) | ^**^ | 31 | 33.95 | (9.61-119.95) | ^**^ |

Supplementary Table 1 (continued) Results of sensitivity analysis showing unadjusted odds ratio (UOR) and 99% CIs of mental health impact from different exposures for all data (n=2,530) and for dataset where missing socio-demographic information has been removed (n=2,180).

|  |  | **Probable anxiety** | | | | | | | | | **Probable depression** | | | | | | | | **Suicidal ideation** | | | | | | | |
| --- | --- | --- | --- | --- | --- | --- | --- | --- | --- | --- | --- | --- | --- | --- | --- | --- | --- | --- | --- | --- | --- | --- | --- | --- | --- | --- |
|  |  | **All records** | | |  | **Records with missing socio-demographic data removed** | | | | **All records** | | | | | **Records with missing socio-demographic data removed** | | | | **All records** | | | | **Records with missing socio-demographic data removed** | | | |
|  |  | **n** | **UOR** | **99%CI** |  | **n** | **UOR** | **99%CI** |  | | **n** | **UOR** | **99%CI** |  | **n** | **UOR** | **99%CI** |  | **n** | **UOR** | **99%CI** |  | **n** | **UOR** | **99%CI** |  |
| **Home of significant other damaged** |  | 285 | 3.32 | (1.59-6.90) | ^**^ | 260 | 3.86 | (1.70-8.77) | ^**^ | | 266 | 3.03 | (1.45-6.31) | ^**^ | 247 | 3.29 | (1.50-7.25) | ^**^ | 118 | 1.72 | (0.72-4.10) |  | 111 | 1.61 | (0.67-3.86) |  |
| **Suburb damaged** |  | 340 | 3.28 | (1.58-6.79) | ^**^ | 303 | 3.78 | (1.67-8.56) | ^**^ | | 327 | 2.88 | (1.42-5.85) | ^**^ | 291 | 3.00 | (1.40-6.40) | ^**^ | 152 | 1.85 | (0.78-4.38) |  | 141 | 1.74 | (0.73-4.13) |  |
| **Non-liveable areas damaged** |  | 251 | 4.09 | (1.96-8.54) | ^**^ | 228 | 4.77 | (2.09-10.87) | ^**^ | | 249 | 3.74 | (1.83-7.65) | ^**^ | 220 | 3.81 | (1.77-8.19) | ^**^ | 117 | 2.36 | (0.99-5.63) |  | 109 | 2.20 | (0.92-5.29) |  |
| **Liveable areas damaged** |  | 151 | 6.07 | (2.85-12.93) | ^**^ | 137 | 7.16 | (3.08-16.63) | ^**^ | | 149 | 5.44 | (2.61-11.34) | ^**^ | 134 | 5.77 | (2.63-12.66) | ^**^ | 70 | 3.27 | (1.33-8.02) | ^*^ | 68 | 3.25 | (1.32-8.01) | ^*^ |
| **Evacuated home** |  | 115 | 6.39 | (2.96-13.79) | ^**^ | 106 | 7.74 | (3.29-18.24) | ^**^ | | 107 | 5.64 | (2.61-12.21) | ^**^ | 97 | 6.10 | (2.66-13.97) | ^**^ | 53 | 3.36 | (1.34-8.43) | ^*^ | 52 | 3.39 | (1.35-8.52) | ^*^ |
| **Displaced <6 months** |  | 60 | 4.29 | (1.91-9.61) | ^**^ | 53 | 5.01 | (2.05-12.29) | ^**^ | | 55 | 3.50 | (1.59-7.75) | ^**^ | 49 | 3.75 | (1.60-8.76) | ^**^ | 34 | 3.09 | (1.18-8.08) | ^*^ | 34 | 3.24 | (1.24-8.50) | ^*^ |
| **Displaced ≥6 months** |  | 45 | 15.00 | (6.09-36.96) | ^**^ | 45 | 18.88 | (7.10-50.24) | ^**^ | | 38 | 10.08 | (4.15-24.47) | ^**^ | 38 | 11.07 | (4.37-28.04) | ^**^ | 18 | 5.19 | (1.77-15.25) | ^**^ | 18 | 5.05 | (1.71-14.87) | ^**^ |
| **Business/farm damaged** |  | 99 | 4.63 | (2.14-10.02) | ^**^ | 88 | 5.32 | (2.25-12.59) | ^**^ | | 89 | 3.78 | (1.77-8.07) | ^**^ | 81 | 4.05 | (1.80-9.10) | ^**^ | 45 | 2.50 | (0.99-6.32) |  | 43 | 2.48 | (0.97-6.31) |  |
| **Evacuated business** |  | 84 | 4.69 | (2.14-10.25) | ^**^ | 73 | 5.33 | (2.23-12.76) | ^**^ | | 77 | 3.89 | (1.81-8.39) | ^**^ | 71 | 4.34 | (1.91-9.84) | ^**^ | 38 | 2.55 | (0.99-6.56) |  | 36 | 2.52 | (0.97-6.54) |  |
| **Cumulative exposure index^†^** | **1** | 33 | 1.06 | (0.45-2.48) |  | 29 | 1.24 | (0.48-3.17) |  | | 38 | 1.14 | (0.50-2.58) |  | 34 | 1.23 | (0.51-2.94) |  | 21 | 0.93 | (0.34-2.56) |  | 19 | 0.87 | (0.31-2.43) |  |
|  | **2** | 87 | 2.38 | (1.10-5.16) | ^*^ | 74 | 2.62 | (1.11-6.21) | ^*^ | | 79 | 1.96 | (0.92-4.19) |  | 67 | 1.94 | (0.86-4.38) |  | 21 | 0.73 | (0.26-1.99) |  | 18 | 0.63 | (0.22-1.78) |  |
|  | **3** | 95 | 2.90 | (1.35-6.27) | ^**^ | 86 | 3.36 | (1.43-7.91) | ^**^ | | 101 | 2.89 | (1.37-6.10) | ^**^ | 91 | 3.02 | (1.36-6.70) | ^**^ | 55 | 2.22 | (0.89-5.53) |  | 49 | 1.97 | (0.79-4.96) |  |
|  | **4** | 116 | 5.74 | (2.66-12.36) | ^**^ | 109 | 6.86 | (2.93-16.09) | ^**^ | | 109 | 4.84 | (2.29-10.22) | ^**^ | 103 | 5.24 | (2.36-11.64) | ^**^ | 46 | 2.58 | (1.02-6.52) | ^*^ | 45 | 2.49 | (0.98-6.32) |  |
|  | **5** | 34 | 13.60 | (5.29-34.98) | ^**^ | 29 | 15.82 | (5.57-44.93) | ^**^ | | 29 | 9.67 | (3.79-24.64) | ^**^ | 24 | 9.67 | (3.52-26.53) | ^**^ | 18 | 7.13 | (2.39-21.27) | ^**^ | 18 | 8.33 | (2.74-25.28) | ^**^ |
